# Exact solutions for the collaborative pickup and delivery problem

**DOI:** 10.1007/s10100-017-0503-x

**Published:** 2017-11-15

**Authors:** Margaretha Gansterer, Richard F. Hartl, Philipp E. H. Salzmann

**Affiliations:** Department for Business Administration, University of Vienna, Oskar-Morgenstern-Platz 1, 1090 Vienna, Austria

**Keywords:** Collaborations, Vehicle routing, Traveling salesman problem, Exact solutions

## Abstract

In this study we investigate the decision problem of a central authority in pickup and delivery carrier collaborations. Customer requests are to be redistributed among participants, such that the total cost is minimized. We formulate the problem as multi-depot traveling salesman problem with pickups and deliveries. We apply three well-established exact solution approaches and compare their performance in terms of computational time. To avoid unrealistic solutions with unevenly distributed workload, we extend the problem by introducing minimum workload constraints. Our computational results show that, while for the original problem Benders decomposition is the method of choice, for the newly formulated problem this method is clearly dominated by the proposed column generation approach. The obtained results can be used as benchmarks for decentralized mechanisms in collaborative pickup and delivery problems.

## Introduction

Horizontal collaboration is a relatively recent phenomenon, where companies at the same level of the supply chain establish partnerships. An example of such a type of collaboration in logistics are carriers who exchange transportation requests in order to increase vehicle fill rates or reduce transportation costs as well as emissions of harmful substances (Beliën et al. 2017). Not surprisingly, collaborative vehicle routing is an active research area of high practical importance (Gansterer and Hartl 2017).

**Fig. 1:**
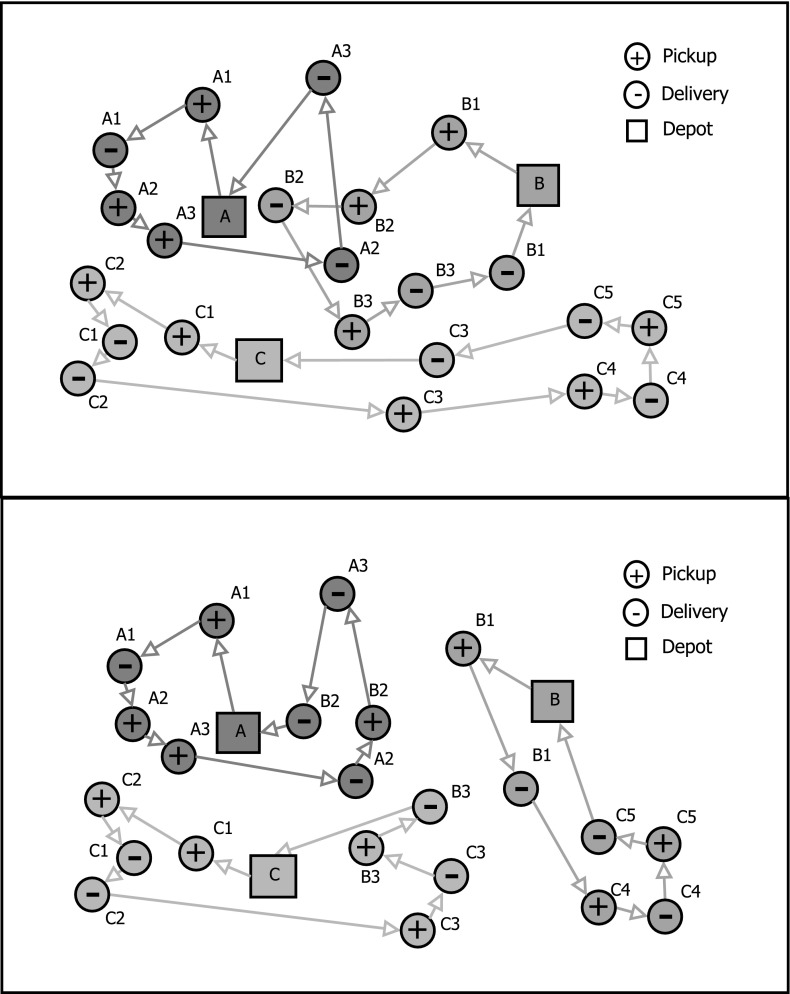
The collaborative pickup and delivery problem of 3 carries (**a**, **b**, **c**). The upper part shows the pre-collaborative setting. An efficient redistribution of customer requests to carriers is shown in the lower part (Gansterer and Hartl 2017)

If collaborative decisions are made by a central authority having full information, this is referred to as *centralized collaborative planning*. An example for such a central authority might be an online platform providing services for collaborative decision making (Dai and Chen 2012). In our study, we focus on such a centralized decision making problem occurring in the less than truckload pickup and delivery market, where customer requests have specified origins and destinations. In this branch of the transportation industry collaborative planning is of particular importance since shipments from different customers can be moved on the same vehicle. This gives carriers much flexibility to share customer requests among each other (Archetti et al. 2014; Gansterer et al. 2016). In Fig. 1 we illustrate the investigated setting with three carriers.

We assume a central authority having full information, aiming at an efficient distribution of customer requests to carriers. The problem has been introduced by Berger and Bierwirth (2010), but no efficient solution techniques have been presented so far. Furthermore, a natural assumption is that carriers are not willing to share *all* their customers. In real-world applications a reasonably even distribution of workload among carriers is a minimum requirement to make collaborative solutions acceptable for competing carriers. Thus, we extend the problem by constraints ensuring that each carrier is assigned a minimum number of customers. We refer to this problem as multi-depot traveling salesman problem with pickups and deliveries (MDTSPPD). For both problem variants (with and without minimum workload constraints), we apply three well-known exact solution methods and compare their performance against a commercial solver. Our computational study shows that Benders decomposition is the method of choice for the original problem formulation. However, if minimum workload constraints are considered, column generation clearly dominates all other solution techniques.

The remainder of the paper is organized as follows. Section 2 provides a literature review. Mathematical models are presented in Sect. 3. We discuss the applied solution methods in Sect. 4. Details on the computational study are presented in Sect. 5. Conclusions and further research are summed up in Sect. 6.

## Literature review

The first studies to systematically assess the potentials of collaborative vehicle routing were presented by Krajewska and Kopfer (2006) and Cruijssen et al. (2007).

A real-world setting of a local courier service of a multi-national logistics company is investigated by Lin (2008). Joint route planning of cooperative carriers is researched by, e.g., Dai and Chen (2012), Buijs et al. (2016), Liu et al. (2010), while Adenso-Díaz et al. (2014), Ergun Ö et al. (2007), Kuyzu (2017) focus on shippers, who want to merge full truckload lanes. The full truckload multi-depot capacitated vehicle routing problem (VRP) in carrier collaboration is presented in Liu et al. (2010).

Several recent studies focus on ecological aspects, like reduced road congestion, noise pollution, and emissions of harmful substances (Montoya-Torres et al. 2016; Pérez-Bernabeu et al. 2015; Sanchez et al. 2016).

In order to approximate optimal solutions even for large real world collaboration problems, many authors propose decomposition strategies. Dai and Chen (2012) use such an approach for a carriers collaborative less than truckload transportation planning problem with pickups and deliveries. Their method consists of two steps. First, a mixed integer programming model, which is a generalization of the lane covering problem, is proposed. Secondly, a set of feasible vehicle tours is constructed. Nadarajah and Bookbinder (2013) also present a two stage framework for less than truckload carrier collaborations. The first stage refers to collaboration between multiple carriers at the entrance to a city, which can be formulated as a VRP with time windows. The second stage involves collaboration between carriers at transshipment facilities. Buijs et al. (2016) study the collaboration between two business units of *Fritom*, a Dutch logistics service provider, and propose alternatives to improve its collaborative transport planning. They introduce the generalized pickup and delivery problem (PDP), which relaxes the common constraints that a load must be transported from its origin to its destination using one vehicle within a single planning period. The authors show different decomposition approaches, which are necessary to solve the real-world instances.

Wang et al. (2014) present a combination of horizontal and vertical carrier collaboration, where both subcontracting and collaborative request exchange are taken into account. Literature reviews on collaborative vehicle routing are presented by Verdonck et al. (2013) and Gansterer and Hartl (2017).

Berger and Bierwirth (2010) introduce the collaborative carrier routing problem, which assigns transportation requests to carriers. The authors come up with two decentralized solution approaches, but no efficient solution method for the centralized problem is presented. Gansterer and Hartl (2016) show that these decentralized mechanisms are particularly powerful if carriers select request based on geographical information. The multiple vehicle VRP in a non-collaborative setting is researched by Lu and Dessouky (2004). As a matter of fact, the authors do not consider workload constraints.

Multi-depot VRP in general are investigated by, e.g., Polacek et al. (2008), Dondo and Cerdá (2007) and Currie and Salhi (2003), while multiple depots and backhaul customers are researched in Salhi and Nagy (1999) and Min et al. (1992). PDP with heterogeneous vehicles are tackled by Irnich (2000). Nagy and Salhi (2005) look at a multi-depot VRP with mixed backhauls and simultaneous pickups and deliveries where a customer can both receive and send goods at the same time. A multi-depot heterogeneous PDP with soft time windows is presented by Bettinelli et al. (2014). Detti et al. (2017) present a multi-depot dial-a-ride problem with heterogeneous vehicles. A survey and typology on multi-depot PDP in multiple regions is provided by Dragomir et al. (2017).

Surveys on pickup and delivery problems are presented by Parragh et al. (2008), Berbeglia et al. (2007), and Berbeglia et al. (2010). Various exact solution methods are applied to this problem class. A branch-and-cut-and-price algorithm for the PDP with shuttle routes is developed by Masson et al. (2014). An extended branch-and-bound algorithm is presented by Kalantari et al. (1985). The PDP with time windows is solved with a column generation scheme by Dumas et al. (1991), a banch-and-cut algorithm by Ropke et al. (2007), and a branch-and-cut-and-price approach by Ropke and Cordeau (2009) and Baldacci et al. (2011). Cherkesly et al. (2016) extend the problem by multiple stacks and solve it using branch-and-price-and-cut, while Cordeau et al. (2010) solve the problem with loading constraints using branch-and-cut. Branch-and-cut as well as branch-and-price are applied by Xue et al. (2016) to the PDP with loading cost. The multiple vehicle PDP is solved using an branch-and-cut algorithm by Lu and Dessouky (2004).

To the best of our knowledge, we are the first to compare different exact solution techniques for the MDTSPPD, and to extend it by the realistic assumption of minimum workload constraints.

## Problem description

The MDTSPPD can be formulated as a routing problem with multiple depots, each used by a single vehicle, i.e. a carrier. The customer requests are paired pickup and delivery requests, meaning that each request is associated with a prespecified origin and destination. The problem belongs to the class of traveling salesman problems with precedence constraints (TSPPC). At each depot, we face the single vehicle case of the VRP with pickups and deliveries (SPDP), which are, according to Parragh et al. (2008), a subclass of VRP with pickups and deliveries (VRPPD). It is classified by Berbeglia et al. (2007) as one-to-one pickup and delivery problem. For the mathematical model we use a Hamiltonian tour formulation as suggested by Lu and Dessouky (2004), where the destination depot of one vehicle is the departure depot of the next vehicle. The model is based on formulations presented in Lu and Dessouky (2004), Gansterer and Hartl (2016) and Berger and Bierwirth (2010):

*n*: number of customers
*m*: number of depots
*P*: set of pickup vertices, $P=\left\{ 1,\ldots ,n\right\}$
*D*: set of delivery vertices, $D=\left\{ n+1,\ldots ,2n\right\}$
*W*: set of depot vertices, $W=\left\{ 2n+1,\ldots ,2n+m+1\right\}$
*N*: set of all vertices, $N=P\cup D\cup W\left\{ 1,\ldots ,2n+m+1\right\}$
*A*: set of all arcs *ij*, $A=N\times N$
$$c_{ij}$$
transportation cost when traveling from *i* to *j*
$$x_{ij}$$
decision variable indicating whether arc *ij* is used or not
$$b_{ij}$$
decision variable indicating whether vertex *i* is visited before vertex *j*

1 $$\begin{aligned}&\min \sum _{ij \in A} x_{ij}c_{ij} \end{aligned}$$

2 $$\begin{aligned}&\quad \sum _{i \in N} x_{ij} = 1 \quad \forall j \in N \end{aligned}$$

3 $$\begin{aligned}&\quad \sum _{j \in N} x_{ij} = 1 \quad \forall i \in N \end{aligned}$$

4 $$\begin{aligned}&\quad b_{ki} \le b_{kj} + (1 - x_{ij}) \quad \forall ij \in A \setminus \{2n+m+1,2n+1\}, k \in N \backslash \{i\} \end{aligned}$$

5 $$\begin{aligned}&\quad b_{kj} \le b_{ki} + (1 - x_{ij}) \quad \forall ij \in A \setminus \{2n+m+1,2n+1\}, k \in N \backslash \{i\} \end{aligned}$$

6 $$\begin{aligned}&\quad x_{ij} \le b_{ij} \quad \forall ij \in A \end{aligned}$$

7 $$\begin{aligned}&\quad b_{ii} = 0 \quad \forall i \in N \end{aligned}$$

8 $$\begin{aligned}&\quad b_{n+i,i} = 0 \quad \forall i \in P \end{aligned}$$

9 $$\begin{aligned}&\quad b_{i, i+n} = 1 \quad \forall i\in P \end{aligned}$$

10 $$\begin{aligned}&\quad b_{ij} = b_{n+i,j} \quad \forall i \in P, j \in W \end{aligned}$$

11 $$\begin{aligned}&\quad b_{i,2n+1} = 0 \quad \forall i \in N \end{aligned}$$

12 $$\begin{aligned}&\quad b_{ij} = 1 \quad \forall i,j \in W\mid {i<j} \end{aligned}$$

13 $$\begin{aligned}&\quad b_{ji} = 0 \quad \forall i,j \in W\mid {i<j} \end{aligned}$$

14 $$\begin{aligned}&\quad b_{i, 2n+m+1} = 1 \quad \forall i \in N \backslash \{2n+m+1\} \end{aligned}$$

15 $$\begin{aligned}&\quad x_{ij} \in \{0,1\} \quad \forall i,j \in N \end{aligned}$$

16 $$\begin{aligned}&\quad b_{ij} \in \{0,1\} \quad \forall i,j \in N \end{aligned}$$

The objective function (1) minimizes the total travel cost. Each vertex has to be entered and left exactly once. This is ensured by constraints (2) and (3). In constraints (4)–(6) we copy the values of the routing decision variables to the precedence decision variables (Lu and Dessouky 2004). Precedences among depots and customers are met by (7)–(14), where constrain (7)–(10) ensure that each pickup node is visited before its associated delivery node, and that customers being assigned to the same depot are served by the same vehicle. In constraint (11) we ensure that no node is visited prior to the first depot. The sequence of depots is determined by constraints (12) and (13). Constraint (14) ensures that the depot $(2n+m+1)$ is the last node in the Hamiltonian tour. While it is necessary to ensure that the routing decision variable $x_{ij}$ is binary, Lu and Dessouky (2004) show that constraint (16) can be relaxed. Subtours are implicitly eliminated by constraints (4)–(6).

*Workload constraints*

In order use the model in the setting of collaborative carriers, it is necessary to include workload limitations. These limitations might be loading quantities or number of customers visited along a tour. Otherwise, in a feasible solution, all requests might be assigned to one single carrier, which will probably not be accepted by the competitors. Thus, we introduce an additional sets of parameters and decision variables:

$$\epsilon _i$$
workload available at customer *i* ($\epsilon _i>0$ at pickup nodes, and $\epsilon _i<0$ at delivery nodes)
$$\overline{R_i}$$
maximum workload for tours at depot *i*
$$\underline{R_i}$$
minimum workload for tours at depot *i*
$$q_i$$
workload when arriving at customer *i*

17 $$\begin{aligned}&q_{j} \le q_{i} + \epsilon _i+M(1-x_{ij}) \quad \forall i \in N \backslash \{2n+m+1\}, j \in N \end{aligned}$$

18 $$\begin{aligned}&q_{j} \ge q_{i} + \epsilon _i-M (1-x_{ij}) \quad \forall i \in N \backslash \{2n+m+1\}, j \in N \end{aligned}$$

19 $$\begin{aligned}&q_{i} - q_{i-1} \le \overline{R_i} \quad \forall i\in W\mid {i>2n+1} \end{aligned}$$

20 $$\begin{aligned}&q_{i} - q_{i-1} \ge \underline{R_i} \quad \forall i\in W\mid {i>2n+1} \end{aligned}$$

21 $$\begin{aligned}&q_i \ge 0 \quad \forall i \in N \end{aligned}$$

Constraints (17) and (18) are required to determine the workload along the route. In the following two constraints, we ensure that a maximum (19) or minimum (20) workload is not violated. If the workload constraint refers to a number of customers that have to be assigned to a depot, $\epsilon _i$ is set to 1 for all pickup nodes $i, i\in P$. Nonnegativity of decision variables $q_i$ is defined by (21).

## Solution methods

In this study, we assess the performance of three different exact approaches, being applied to the MDTSPPD. These approaches are (i) branch-and-cut, (ii) benders decomposition, and (iii) column generation. Benchmark results are generated using a standard optimization software (CPLEX Optimizer 12.7).^1^

### Branch-and-cut

The branch-and-cut algorithm is an extension of the well known branch-and-bound approach. The main difference is the way solutions on nodes of the search tree are processed. In a branch-and-cut algorithm, additional constraints are used to strengthen the linear programming relaxation if required. These cuts do not exist in the original problem definition. A similar approach are *lazy constraints*. Lazy constraints are part of the problem definition, but in the branch-and-cut search, they are only added if required. If a candidate solution is found, the algorithm checks if it is feasible with respect to the lazy constraints. A violated constraint is added, and the solution gets re-evaluated. We use the procedure proposed by Lu and Dessouky (2004), including the following cuts (Lu and Dessouky 2004; Ropke and Cordeau 2009):

*Transfer constraints* The following valid inequalities hold for an arbitrary collection of nodes $(h_1, \dots , h_k) \in N\backslash \{i, 2n+m+1\}, 1 \le k \le |N|-2$ (Lu and Dessouky 2004):

22 $$\begin{aligned} b_{n+i, h_1} + \sum _{j=1}^{k-1} b_{h_j, h_{j+1}} + b_{h_k,i} \le k \end{aligned}$$

*Adjacent constraints* These valid inequalities strengthen the precedence constraints by checking the requirements for pairs of directly connected nodes (Lu and Dessouky 2004). Whenever a pickup node *i* is visited before some node *k*, the corresponding delivery node ($i+n$) has to be visited after *k*.

23 $$\begin{aligned} b_{ki} + b_{k,i+n}\ge & {} x_{i,k} + x_{k,i} \quad \forall i \in P, k \in N \backslash \{ i, i+n \} \end{aligned}$$

24 $$\begin{aligned} b_{ik} + b_{i+n,k}\ge & {} x_{i+n,k} + x_{k,i+n} \quad \forall i \in P, k \in N \backslash \{ i,i+n \} \end{aligned}$$

*Pairing constraints* A delivery node has to have more preceding nodes than its associated pickup node (Lu and Dessouky 2004):

25 $$\begin{aligned} \sum _{k \in N} b_{ki} + 1 \ge \sum _{k\in N} b_{k, i+n} \quad \forall i \in P \end{aligned}$$

*Demand constraints* In paired PDP it can be assumed that the demand at the delivery node is equal to the supply at the pickup node. Making use of this characteristic, Lu and Dessouky (2004) present a cut that can be used even if the real demand *values* are not known. The idea is that only very few combinations will sum up to zero (which is always the case for a specific pickup and delivery pair). Thus, if demands are not known, an artificial demand is assigned to each of the customers. This demand has to be unique and benefits from being not constructable from other demands. The easiest way to determine such sets of demands is to use prime numbers. For cutting, we sum up all demands served by a given depot:

26 $$\begin{aligned} \sum _{k \in N} b_{ki} d_k = 0 \quad \forall i \in W, \end{aligned}$$

where $d_k$ is the demand of customer *k* (it is assumed that $d_k> 0$ if *k* is a pickup node, and $d_k< 0$ if *k* is a delivery node).

In the proposed branch-and-cut approach, precedence constraints (4)–(6) are used as lazy constraints. Since in the problem formulation, precedence constraints are implicitly used to eliminate subtours, it seems to be beneficial to use additional subtour elimination constraints as lazy constraints ($S\subseteq N$, $\emptyset \ne S \ne N$):

27 $$\begin{aligned} \sum _{e \in E(S)} x_e \le |S| - 1, \end{aligned}$$

where $E(s):=\left\{ e\in E:\left| e\cap S\right| =2\right\}$. These lazy constraints will enforce that the sum of connections within each subset of nodes *S* is smaller than the size of the subset.

### Benders decomposition

As a second approach, we embed Benders Decomposition (Benders 1962) into the branch-and-cut procedure. This approach is described in, e.g., Sridhar and Park (2000). In each node of the branch-and-cut tree, Benders Decomposition is applied to the linear relaxations. The general concept is to decompose the problem into smaller subproblems, called stages. Each stage contains a set of variables and constraints of the original problem. The stages are solved iteratively. Once a solution of the first stage is determined, the second stage is solved using the solution of the first stage. As long as the first stage solution leads to an infeasible second stage solution, new constraints are added to the first stage master problem. These iteratively added constraints are called Benders feasibility cuts. The optimal solution is found, if the first stage solution leads to a valid second stage solution and no further cuts are required.

The original problem (see Sect. 3) has two types of decision variables: one for the routing decisions and one for the precedence relations. Since the latter restricts the routing decisions, it seems reasonable to use them for the second stage problem, while the first stage generates candidate solutions. However, these routes do not incorporate the precedence constraints (6)–(14), since these are in the second stage, and may therefore contain subtours. Therefore, the second stage ensures that solutions that contain subtours and violate precedence constraints are withdrawn from the solution space (cf. Sexton and Bodin 1985a, b; Contardo and Martinelli 2014). The constraints of the proposed model extensions (see Sect. 3) are added to the first stage.

### Column generation

In column generation, the decision problem is decomposed into a master- and a subproblem. While the number of constraints is fixed, the number of decision variables (columns) increases over time. For the MDTSPPD, we separate the routing decision (subproblem) from the route selection (master problem), which is a linear relaxation of the following set partitioning problem:

$$\gamma $$
set of valid routes (according to the problem definition in Sect. 3)
$$c_{\gamma , d}$$
the costs of route $\gamma$ when serviced by depot *d*
$$\alpha _{i,\gamma }$$
a constant indicating if customer *i* is on route $\gamma$
$$y_{\gamma , d}$$
decision variable indicating if route $\gamma$ is in the solution and if depot *d* is used to service the route

28 $$\begin{aligned}&\min \sum _{\gamma \in \Gamma , d \in W} y_{\gamma ,d} c_{\gamma ,d} \end{aligned}$$

29 $$\begin{aligned}&\quad \sum _{\gamma \in \Gamma , d \in D} y_{\gamma , d}\alpha _{i,\gamma } = 1 \quad \forall i \in N\setminus W \end{aligned}$$

30 $$\begin{aligned}&\quad \sum _{\gamma \in \Gamma } y_{\gamma , d} = 1 \quad \forall d \in W \end{aligned}$$

31 $$\begin{aligned}&\quad y_{\gamma , d} \in \{0,1\} \quad \forall \gamma \in \Gamma , d \in W \end{aligned}$$

The objective function (28) minimizes the total routing costs, while constraint (29) ensures that each request is serviced by a route. Each depot has to be assigned to at least one route (30). The binary property of the decision variables are defined in (31).

As part of an iterative process, the proposed set partitioning formulation (master problem) will serve two purposes: (i) selecting a set of routes that ensure that every request is serviced, and (ii) updating the dual costs of each request and depot. A shortest path problem based on the properties of the vehicle flow formulation (Sect. 3) generates new promising routes based on the updated dual information provided by the master problem. This iterative process is performed until the subproblem is not able to identify additional routes that could reduce the objective function of the master problem. Dominance rules can be used to decrease the number of routes in the subproblem.

For the subproblem, we apply a labeling algorithm, where we start from a depot and gradually expand the route by a new node or customer. Every time doing so we have to check the feasibility (i.e. capacity restrictions) of the newly created route. Every time a route gets extended the new and the existing routes are compared based on the dominance rules (see below). Dominated routes get discarded. In our algorithm, we deal with all depots at once. By introducing an artificial depot with a distance of zero to each of the customers, the shortest path problem with pickup and delivery can be used (e.g. Desrosiers and Dumas 1988). Every route that gets extended to a depot, has to be extended to each of the depots, and after being checked for dominance, added to the master problem.

We apply dominance rules proposed by Ropke and Cordeau (2009), and Contardo and Martinelli (2014) for multi-depot VRP:

32 $$\begin{aligned} x.{ costs} \le&{ y.costs} \end{aligned}$$

33 $$\begin{aligned} x.{ nodesVisited} \supseteq&{ y.nodesVisited} \end{aligned}$$

34 $$\begin{aligned} x.{ openRequests} \subseteq&{ y.openRequests} \end{aligned}$$

35 $$\begin{aligned} x.{ lastNodeOfTour} =&{ y.lastNodeofTour} \end{aligned}$$

36 $$\begin{aligned} x.{ depotUsed} =&{ y.depotUsed} \end{aligned}$$

A route *x* dominates a route *y* if it is cheaper, and visits at least the same nodes. Furthermore, we use *open requests*, i.e requests where the pickup but not the delivery node is visited, as additional dominance criterion. All criteria have to be met for a pair of routes having the same starting and ending node.

## Computational results

For our computational study we use data developed by Berger and Bierwirth (2010). The authors present three instance sets which refer to different degrees of competition between the carriers: (i) adjacent (ii) overlapping, and (iii) identical customer regions. For each scenario (A, O, and I), there are 30 instances, with 3 depots and 9 transportation requests. For all computational experiments, we limit the runtime to 30 min. The algorithms are run single-threaded on an Intel Xeon CPU with 2.50 GHz. It should be noted that Berger and Bierwirth (2010) set their time limit to 120 min on a PC P4 with 2800 MHz.

### Original problem

In the first part of our computational study, we assess the solution methods being applied to the original problem, i.e. without the additional constraints (see Sect. 3) on minimum workload.

In Sect. 4, we presented three variants of the Branch-and-cut algorithm, which are (i) with lazy constraints (*LC*), (ii) with lazy constraints and subtour elimination (*LS&SE*), (iii) without lazy constraints (*woLC*). As a first step, we want to investigate the necessity of including the proposed lazy constraints. The results are presented in Table 1.

**Table 1:** Average runtimes (in s) of CPLEX and of the 3 proposed branch-and-cut variants being applied to test instances A, I, and O

Instance	CPLEX	LC	LC&SE	woLC
A	257.04	341.93	332.73	100.28
I	434.36	599.67	599.14	183.73
O	677.86	850.27	747.19	661.71
Average	456.42	597.29	559.69	315.24
#Unsolved	11	16	15	11

From the average runtimes we see that variant *woLC* outperforms the other two. For all three scenarios, this methods requires the minimum average runtime, and is able to solve the maximum number of instances. The reason for this is that the lazy constraints are rarely binding constraints. Thus, for all remaining tests, we only use the *woLC* configuration for the branch-and-cut algorithm. Not surprisingly, instances O require the longest runtimes, since the solution space increases with the degree of competition.

In Table 2 we compare Branch-and-cut, Benders decomposition, and column generation against CPLEX.

**Table 2:** Average runtimes (in s) of CPLEX and of Branch-and-cut (*Bac*), Benders decomposition (*BD*), and column generation (*CG*) for test instances A, I, and O

Instance	CPLEX	Bac	BD	CG
A	257.04	100.28	27.35	>1800
I	434.36	183.73	179.42	>1800
O	677.86	661.71	434.02	>1800
Average	456.42	315.24	213.60	$>1800$
#Unsolved	11	11	4	90

The last line reports the number of instances that could not be solved within the given time limit of 30 min

The results show that Benders decomposition outperforms all other approaches. For each of the test scenarios, this methods needs the lowest computational time to reach the optimal solution, while there are only 4 instances (out of 90) that could not be solved within the given time limit of 30 min. It should be noted that the proposed column generation approach cannot solve any of the instances within the time limit of 30 min. This can be explained by the scarcely constrained solution space, which is disadvantageous for column generation-based methods. In the second part of our computational study (see Sect. 5.2), we see that additional constraints are a boost for the column generation approach.

### Workload constraints

In the second part of our computational study, we use the extended model presented in Sect. 3, where each carrier requires a minimum workload. In Table 3 we show the increase in total cost, depending on the degree of required workload.

**Table 3:** Total costs (optimal solution) with different limits on the required workload per carrier: no limit (*no*), each carriers has to keep 1 customer (*1cust*), each carrier has to keep 2 customers (*2cust*)

Instance	no	1cust (%)	2cust (%)
A	270.49	12.64	21.87
I	263.00	17.41	28.49
O	267.49	24.91	45.52
Average	267.00	18.32	31.96

For *cust1* and *cust2* we report the percentage increase compared to *no*

The results show that the inclusion of a minimum workload constraint of 1 customer increases the total costs on average by 18.32%. If each carrier has to keep at least 2 of the initial customers, the average cost increase is more than 30%. This is in line with the literature on collaborative vehicle routing, where several studies show that centralized solutions yield up to 30% higher collaboration profits than decentralized solutions (Gansterer and Hartl 2017; Cruijssen et al. 2007; Montoya-Torres et al. 2016; Lin 2008).

In Table 4 we present the average runtimes needed to solve the problem with workload constraints.

**Table 4:** The upper part reports the average runtimes for each method, i.e. CPLEX, Branch-and-cut (*Bac*), Benders decomposition (*BD*), and column generation (*CG*), needed to solve the problem with 3 variants of workload constraints: no limit (*no*), each carriers has to keep 1 customer (*1cust*), each carrier has to keep 2 customers (*2cust*)

Workload	CPLEX	Bac	BD	CG
	Runtimes			
No	456.04	315.24	213.60	$>1800$
1cust	1286.44	1023.72	432.72	$>1800$
2cust	1689.62	1023.00	1056.87	566.55
	#unsolved			
No	11	11	4	90
1cust	51	40	12	90
2cust	80	41	43	0

The lower part lists the number of instances that could not be solved within the given time limit (30  min)

In case of low or no workload limits, Benders decomposition is still the method of choice. However, if carriers have to keep more than one of their customers, column generation finds the optimal solutions much faster. Also the number of instances that could not be solved within the given time limit of 30 min, clearly depends on the workload constraint. If there is a strong restriction on the number of customers each carrier has to keep, column generation finds all optimal solutions within very short amount of time, while Benders decomposition fails in 43 (out of 90) instances. In Table 5 we provide more detailed results on the setting, where carriers have to keep 2 of their customers (2*cust*).

**Table 5:** Average runtimes for each method, i.e. CPLEX, branch-and-cut (*Bac*), benders decomposition (*BD*), and column generation (*CG*), needed to solve problem variant *2cust*, where each carrier has to keep 2 customers

Instance	CPLEX	Bac	BD	CG
A	1494.48	382.74	484.66	596.05
I	1793.03	1256.32	1099.21	593.51
O	1781.33	1429.95	1586.76	510.09
Average	1689.62	1023.00	1056.87	566.55

We see that column generation shows a very strong performance for instance sets with a high degree of competition (I and O). For these instances, this method finds the optimal solutions about 3 times as fast as the second best method. This is not very surprising, since it is well-known that column generation takes advantage of solution spaces with few valid solutions. However, it is remarkable that even instance set *O* can be solved without any loss in performance. This makes column generation a very powerful method for problems with a high degree of competition.

Hence, we can conclude that for the original problem proposed by Berger and Bierwirth (2010), Benders decomposition is the method of choice, while for the newly introduced setting, column generation should be preferred.

## Conclusion

In this study we investigated a decision problem faced by a centralized decision maker in carrier collaborations. Pickup and delivery requests are to be redistributed among participants, such that the total cost is minimized. This problem was formulated as MDTSPPD. Three well-established exact solutions approaches were compared in terms of their computational performance.

To avoid unrealistic solutions with unevenly distributed workload, we extend the problem by minimum workload constraints. Our computational results show that, while for the original problem Benders decomposition is the method of choice, for the newly formulated problem this method is clearly dominated by the proposed column generation approach.

We showed that the proposed minimum workload constraints have a surprisingly strong impact on the total costs. If carriers want to keep a minimum workload of at least 30% of their initial one, the total costs increase on average by 18.32%. If 60% of the initial customers have to remain unchanged, the average cost go up by more than 30%.

The results of the computational study can be used as benchmarks for decentralized mechanisms in collaborative PDP problems. The insights on the performance of the investigated methods are useful for generating results for similar test cases.

## Footnotes

1
https://www-01.ibm.com/software/commerce/optimization/cplex-optimizer/.
